# The bidirectional longitudinal association between health-related quality of life and academic performance in adolescents: DADOS study

**DOI:** 10.1007/s11136-022-03291-z

**Published:** 2022-11-16

**Authors:** Mireia Adelantado-Renau, Irene Monzonís-Carda, Diego Moliner-Urdiales, Maria Reyes Beltran-Valls

**Affiliations:** LIFE Research Group, Faculty of Humanities and Social Sciences, Department of Education and Specific Didactics, Universitat Jaume I, Av. Vicent Sos Baynat, S/N, 12071 Castellon, Spain

**Keywords:** Well-being, Health, Academic achievement, Adolescence

## Abstract

**Purpose:**

Although previous evidence has suggested a relationship between health-related quality of life (HRQoL) and academic performance, the directionality of this association is understudied and remains to be clarified. Thus, the primary objective of this study was to explore the bidirectional association between HRQoL and academic performance in adolescents between two timepoints with a 24-month interval. A secondary aim was to analyze whether this association varies between boys and girls.

**Methods:**

This is a bidirectional longitudinal analysis with 257 adolescents (13.9 ± 0.3 years at baseline) from the DADOS study. HRQoL was measured using the KIDSCREEN-10 questionnaire. Academic performance was assessed through academic grades and the Spanish version of the Science Research Associates Test of Educational Ability.

**Results:**

Cross-lagged analyses revealed that HRQoL at baseline was not associated with academic performance 24 months later, while all the academic grades and the overall score of academic abilities at baseline were positively associated with HRQoL at follow-up in adolescents. Results of the stratified analyses by sex were largely similar. Specifically, in girls, math, language, physical education, and grade point average at baseline were positively associated with HRQoL 24 months later, while in boys, all the academic grades indicators (except physical education), numeric ability, and the overall score of academic abilities at baseline were positively associated with HRQoL at follow-up.

**Conclusion:**

These findings suggest that academic performance in early adolescence may predict HRQoL 24 months later. Health and education professionals could benefit from collaborating to achieve both improved academic performance and HRQoL in youth.

**Supplementary Information:**

The online version contains supplementary material available at 10.1007/s11136-022-03291-z.

## Introduction

Well-being is as a higher-order construct that integrates health (e.g., mental or physical functions), health-related (e.g., work or habits), and non-health-related domains (e.g., autonomy or integrity), which depends on complex interactions between individual and contextual factors [1]. Indeed, prior research has shown that individuals with high well-being are usually successful across multiple domains of life, including work and social relationships, and show better mental and physical health, as well as longer longevity [2]. Well-being encompasses some related but distinguishable constructs such as health-related quality of life (HRQoL), which could be defined as a broad multidimensional concept comprising the individual's perceptions of how life is going in terms of health and health-related domains [1]. HRQoL is considered a relevant health indicator, since it allows to capture the burden of disease from the perspective of an individual, and has been proposed as a predictor of mortality and morbidity [3]. From a public health perspective, assessing HRQoL during adolescence is of paramount importance, since quality of life in this period stablishes the basis for quality of life and health status in adulthood [4]. Thus, identifying factors related to adolescents’ HRQoL is of relevance to advance in the understanding of this multidimensional construct.

It has been recently suggested that HRQoL could be positively related with academic performance [5], which refers to educational goals that students have to reach in a particular period of time [6]. Importantly, academic performance may strongly shape a person’s life chances in terms of work and health. In fact, higher academic performance during adolescence has been associated with better earnings [7] and health status later in life [8]. While the relationship of adolescents’ health with HRQoL [9, 10] and academic performance has been widely investigated [6], how the psychological construct of HRQoL and academic performance are associated is understudied and remains to be clarified.

Regarding the overall construct of well-being and its association with academic performance, a recent systematic review conducted by Amholt et al. [11] concluded that findings from previous studies were inconsistent, since both positive and null associations were reported. Moreover, the studies included in this review, mostly with a cross-sectional design, focused on analyzing the unidirectional relationship between subjective well-being and students’ academic performance [11]. Interestingly, few previous research investigated the plausibility of a bidirectional association between these two constructs in adolescents, which suggested that the direction of this association is still unknown since divergent results were reported [12–14]. For instance, Wu et al. [12] investigated the bidirectional association between the main components of subjective well-being (i.e., life satisfaction, and positive and negative affect) and a standardized index of academic performance, showing that only life satisfaction and positive affect at baseline were positively correlated with adolescents’ academic performance 14 months later. Conversely, Steinmayr et al. [13] reported that adolescents’ grade point average (GPA) at baseline was positively associated with changes in life satisfaction at 1-year follow-up, while there was no association in the other direction. Additionally, Ng et al. [14], who examined the association between life satisfaction and GPA in two waves with a 5-month interval in a sample of adolescents, suggested a positive reciprocal causal relationship. Regarding the specific construct of HRQoL, there is only one study that has examined its bidirectional association with academic performance in adolescents, which reported controversial results and gender differences [15]. Specifically, Bortes et al. [15] found a positive association between HRQoL at baseline and GPA 24 months later, and a negative association between GPA at baseline and HRQoL at follow-up in adolescent girls, while no associations were found among boys.

The scarce literature examining the bidirectional longitudinal association between HRQoL and academic performance evidenced two major limitations. First, most previous studies included only a global indicator of academic performance, without examining the association of each single academic performance indicator with HRQoL. Moreover, in this context, prior evidence did not include academic abilities, which reflect competences and content acquired in specific areas of knowledge. Second, most of these studies present a relatively short-term longitudinal design. Therefore, given the paucity of knowledge and the lack of conclusive findings, more studies are necessary to address these gaps in the literature and to clarify the directionality of the association between HRQoL and academic performance in adolescents. Thus, the present study intended to explore the bidirectional association between HRQoL and academic performance in adolescents between two timepoints with a 24-month interval. Since the influence of sex in this association remains unclear, a secondary aim was to analyze whether this association varies between boys and girls.

## Methods

### Study design and sample selection

This study is part of the DADOS (Deporte, ADOlescencia y Salud) research project, a 3-year longitudinal study aimed to examine the influence of lifestyle behaviors on health and academic performance during adolescence. The results presented in this study belong to baseline (obtained between February and May of 2015) and follow-up data (obtained between February and May of 2017). A convenience sampling technique was used to recruit participants. For that purpose, advertising leaflets about the research project were sent to secondary schools and sport clubs located in the province of Castellon (Spain), which included main information about the aim and the study protocol. The inclusion criteria were to be enrolled in second grade of secondary school, and not to be diagnosed of any physical (i.e., locomotor system injury) or mental (i.e., intellectual disability) impairment. Volunteers who met the inclusion criteria were included in the study. A total of 257 adolescents (121 girls) aged 13.9 ± 0.3 years at baseline with valid data for HRQoL and academic performance at baseline were included in the analyses.

Adolescents and their parents or guardians were informed of the nature and characteristics of the study, and all of them provided a written informed consent. The DADOS study protocol was designed in accordance with the ethical guidelines of the Declaration of Helsinki 1964 (last revision of Fortaleza, Brazil, 2013) and approved by the Research Ethics Committee of the University Jaume I of Castellon (Spain).

### Health-related quality of life

HRQoL was assessed with the KIDSCREEN-10 questionnaire, a valid and reliably scale to analyze HRQoL among youth population [16]. The reliability and validity of the questionnaire have been examined previously in adolescents showing good reliability (Cronbach’s α = 0.82) and criterion validity (r = 0.91) [16]. Similar reliability results have also been obtained in the current study (Cronbach’s α = 0.77). This questionnaire consists of 10 items rated in a 5-point Likert scale (i.e., 1 = “not at all”, 2 = “slightly”, 3 = “moderately”, 4 = “very”, and 5 = “extremely”). For each item, responses were coded so that higher values indicate better HRQoL. Then, the sum of the items was calculated, and it was transformed based on the RASCH-Person parameters estimates [17]. A higher score in the questionnaire indicates better HRQoL.

### Academic performance

Academic performance was assessed through the final academic grades from the first (13 years) and the third (15 years) grade of secondary school, provided by each school’s secretary office. They are based on a ten-point scale (0 indicates the lowest achievement and 10 indicates the highest achievement) and can be classified in the following categories: unsatisfactory (0 to 4.9), satisfactory (5 to 5.9), good (6 to 6.9), very good (7 to 8.9), and excellent (9 to 10). GPA score and individual grades for the following subjects were included in the analyses: natural sciences, social sciences, math, language, and physical education. GPA score was defined as the average of the scores achieved by students in all subjects.

The Spanish version of the Science Research Associates Test of Educational Ability (TEA) was used to measure academic abilities [18]. This test provides general measures of three areas of intelligence and skills of learning: verbal (i.e., command of language), numeric (i.e., speed and precision in performing operations with numbers and quantitative concepts), and reasoning (i.e., the skill to find logical order in sets of numbers, figures, or letters). Scores for the three areas were obtained by adding positive answers. Overall score was calculated by adding the three areas scores (i.e., verbal, numeric, and reasoning). Based on the age range of our sample, level three of the TEA questionnaire designed for ages 14 to 18 years was used in both assessments (reliability: verbal α = 0.74, numeric α = 0.87, reasoning α = 0.77, and overall score α = 0.89).

### Covariates

Sex, pubertal stage, waist circumference, socioeconomic status, and parents’ education level were included as covariates in the statistical analyses due to their relationship with the study variables [6, 19, 20].

*Pubertal stage.* Pubertal stage was self-reported according to the five stages described by Tanner and Whitehouse [21]. It is based on external primary and secondary sexual characteristics, which are described by the participants using standard pictures according to Tanner instructions.

*Waist circumference.* Waist circumference was measured twice to the nearest 1 mm with a non-elastic tape applied horizontally midway between the lowest rib margin and the iliac crest, at the end of gentle expiration with the adolescent in a standing position. The average measure was used for the analyses.

*Socioeconomic status.* The Family Affluence Scale (FAS) developed by Currie et al. [22] was used as a proxy of socioeconomic status (ranging from 0 to 8), which is based on material conditions in the family such as car ownership, bedroom occupancy, computer ownership, and home internet access.

*Parents’ education level*. Parents or legal guardians reported their education level which was categorized into two groups using the highest education level obtained by the mother or the father: (i) below university education, and (ii) university education.

### Statistical analysis

Descriptive characteristics are presented as mean and standard deviations or percentages. All variables were checked for normality using both graphical (normal probability plots) and statistical (Kolmogorov- Smirnov test) procedures.

**Fig. 1:**
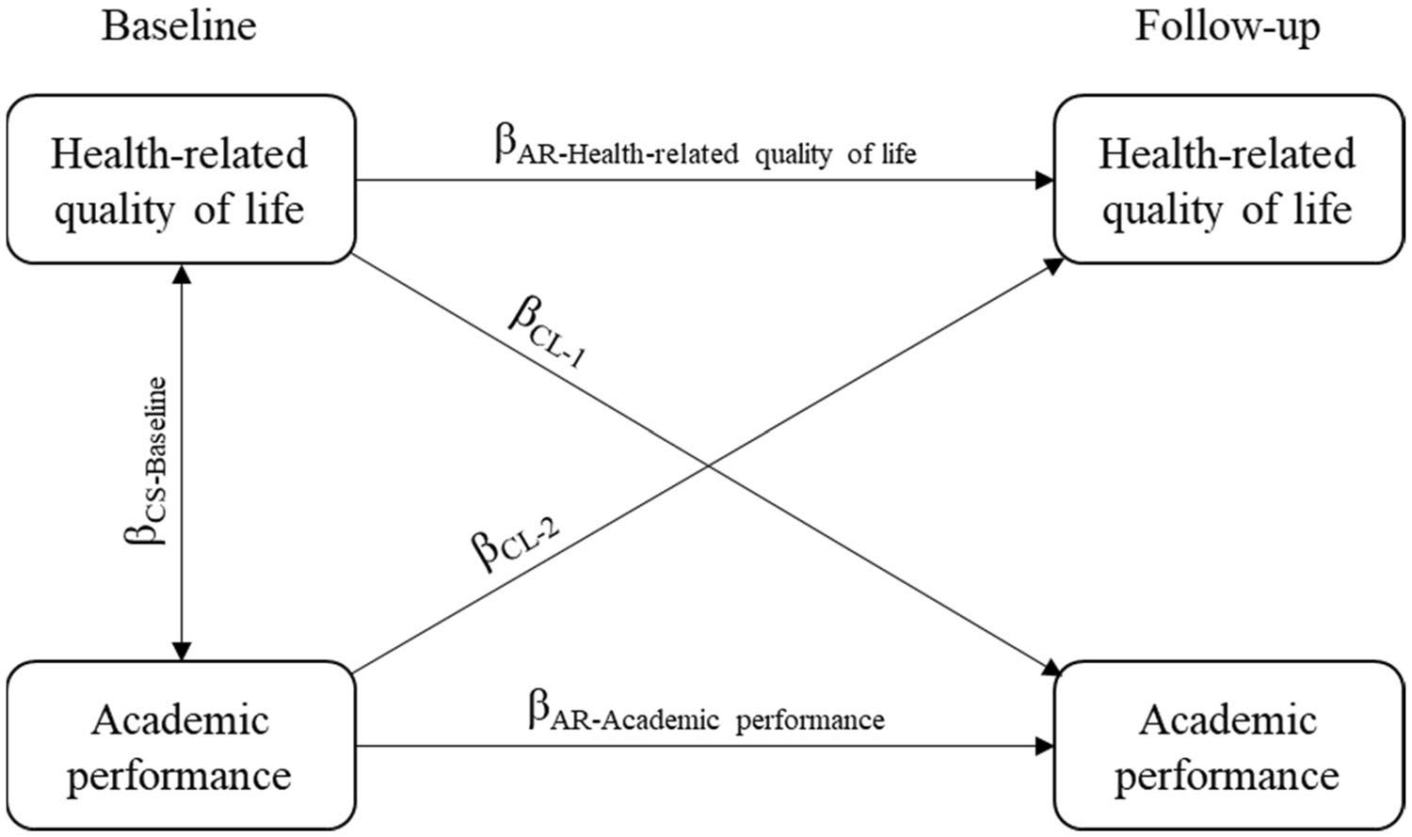
Visualization of cross-lagged panel modeling approach. *AR* Autoregressive, *CL* Cross-lagged path, *CS* Cross-sectional path

The bidirectional association between HRQoL and academic performance indicators was examined using a cross-lagged modeling approach, through the Lavaan package in R [23]. A depiction of the general cross-lagged panel model is presented in Fig. 1. In these path analyses, all associations were adjusted for each other: that is to say, analyses are adjusted for the mutual prospective associations that represent the bidirectional associations between HRQoL and academic performance indicators (the cross-lagged pathways β_CL-1_ and β_CL-2_), the cross-sectional paths (β_CS-Baseline_), and the underlying associations of HRQoL over time (autoregressive path, β_AR-Health-related quality of life_) and academic performance indicators over time (autoregressive path, β_AR-Academic performance_). The autoregressive paths describe the stability of individual differences in the measured variables from baseline to follow-up. A small (closer to zero) autoregressive coefficient indicates less stability, while a larger (closer to 1) autoregressive coefficient indicates more stability of the variable over time [24].

Models were assessed using several fit indexes and information criteria: the Comparative Fit Index (CFI) and Tucker-Lewis Index (TLI), which should both be close to or exceed 0.95, the Root Mean Square Error of Approximation (RMSEA), which should be close to or below 0.06, and the Standardized Root Mean square Residual (SRMR), which should be close to or below 0.08 [25]. All models were adjusted for sex, pubertal stage, waist circumference, socioeconomic status, and parents’ education level.

The Full Information Maximum Likelihood Estimator was implemented to preserve all available data (*n* = 257) [23]. This estimator is considered a standard approach to prevent listwise deletion of participants with missing data [23]. In these analyses, we used false discovery rate based on the Benjamini–Hochberg method to adjust for multiple comparisons. Briefly, this method uses ranked p-values to determine the cut-off, at which point the Type-I error rate is below 0.05 [26]. Lastly, several sensitivity analyses were performed. Firstly, to ensure that the results were not biased due to the estimation of missing data from the second timepoint, analyses were conducted only including the participants with complete HRQoL and academic performance data at both timepoints (*n* = 184). Secondly, stratified analyses by sex were conducted for the cross-lagged paths in which associations were statistically significant. All the statistical analyses of this study were performed using SPSS v. 27 (IBM Corp., Armonk, NY) and R version 3.6.0 using the Lavaan package (The R foundation for Statistical Computing, Vienna, Austria), and the level of significance was set at *p* < 0.05.

## Results

The characteristics of the adolescents at baseline and at 24-month follow-up are shown in Table 1. Participants’ HRQoL score was 50.1 at baseline and 48.7 at follow-up. Academic grades ranged from 6.9 to 8.1 at baseline and from 6.3 to 8.2 at follow-up. Regarding academic abilities, the overall score was 49.0 at baseline and 59.8 at follow-up.

**Table 1:** Descriptive characteristics of participants at baseline and at 24-month follow-up

	Baseline	Follow-up
*n*	257	184
Age (years)	13.9 ± 0.3	15.8 ± 0.3
Pubertal stage (II–V) (%)	8/35/47/10	0/11/52/37
Waist Circumference (cm)	67.4 ± 5.9	71.4 ± 6.3
Socioeconomic status (0–8)	4.2 ± 1.4	–
Parents with university-level education (%)	48.6	–
Health-related quality of life	50.1 ± 8.1	48.7 ± 6.4
Academic performance		
Academic grades (0–10)		
Natural Sciences	7.1 ± 1.6	6.6 ± 1.5
Social Sciences	7.1 ± 1.6	6.9 ± 1.8
Math	6.9 ± 1.6	6.3 ± 1.8
Language	7.0 ± 1.5	6.3 ± 1.6
Physical Education	8.1 ± 1.1	8.2 ± 1.2
Grade point average	7.2 ± 1.3	6.7 ± 1.3
Academic abilities		
Verbal ability (0–50)	18.8 ± 5.3	22.4 ± 5.7
Numeric ability (0–30)	13.5 ± 4.8	16.5 ± 5.2
Reasoning ability (0–30)	16.6 ± 5.7	20.9 ± 4.9
Overall score (0–110)	49.0 ± 12.6	59.8 ± 12.4

Data are presented as mean ± standard deviation or percentages

Bidirectional longitudinal associations between HRQoL and academic performance based on the cross-lagged panel models after adjustment for sex, pubertal stage, waist circumference, socioeconomic status, and parents’ education level are shown in Table 2. The headings used in the table are represented as pathways in Fig. 1. At baseline, after multiple comparisons correction, only GPA was cross-sectionally and positively associated with HRQoL (*p* = 0.010). HRQoL at baseline was not associated with any of the academic performance indicators 24 months later (all *p* > 0.05). Nevertheless, all the academic grades and the overall score of academic abilities at baseline were positively associated with HRQoL at follow-up (all *p* < 0.05).

**Table 2:** Bidirectional associations between health-related quality of life and academic performance based on the cross-lagged panel models (*n* = 257)

	HRQoL → AP			AP → HRQoL			Cross-sectional			Fit measures	
	β_CL-1_	*p*	*p*_FDR_	β_CL-2_	*p*	*p*_FDR_	β_CS-Baseline_	*p*	*p*_FDR_	CFI	RMSEA
Health-related quality of life											
*Academic grades*											
Natural Sciences	0.003 (− 0.088, 0.094)	0.947	0.947	0.171 (0.061, 0.282)	**0.004**	**0.008**	0.130 (0.027, 0.234)	**0.015**	0.050	0.965	0.059
Social Sciences	0.077 (− 0.043, 0.198)	0.209	0.523	0.181 (0.061, 0.300)	**0.004**	**0.008**	0.106 (0.000, 0.212)	0.056	0.093	0.986	0.034
Math	− 0.123 (− 0.226, − 0.020)	**0.019**	0.190	0.238 (0.124, 0.352)	** < 0.001**	** < 0.001**	0.121 (0.016, 0.226)	**0.026**	0.065	0.973	0.048
Language	− 0.026 (− 0.131, 0.079)	0.625	0.781	0.214 (0.108, 0.320)	** < 0.001**	** < 0.001**	0.132 (0.031, 0.234)	**0.011**	0.050	0.965	0.057
Physical Education	0.106 (− 0.030, 0.242)	0.137	0.523	0.156 (0.043, 0.270)	**0.007**	**0.012**	0.120 (0.008, 0.232)	**0.037**	0.074	1.000	0.000
GPA	− 0.014 (− 0.094, 0.066)	0.733	0.814	0.205 (0.091, 0.320)	**0.001**	**0.003**	0.167 (0.069, 0.264)	**0.001**	**0.010**	0.975	0.055
*Academic abilities*											
Verbal ability	− 0.049 (− 0.165, 0.067)	0.410	0.683	0.106 (− 0.013, 0.225)	0.082	0.091	0.113 (− 0.009, 0.235)	0.066	0.094	1.000	0.000
Numeric ability	− 0.074 (− 0.181, 0.034)	0.176	0.523	0.124 (− 0.017, 0.265)	0.080	0.091	0.088 (− 0.019, 0.196)	0.112	0.140	0.966	0.060
Reasoning ability	0.037 (− 0.068, 0.142)	0.492	0.703	0.111 (− 0.018, 0.240)	0.096	0.096	0.023 (− 0.102, 0.148)	0.719	0.719	0.999	0.009
Overall score	− 0.038 (− 0.126, 0.051)	0.403	0.683	0.146 (0.014, 0.278)	**0.030**	**0.043**	0.091 (− 0.031, 0.212)	0.144	0.160	0.987	0.037

Results showed as standardized coefficients and 95% confidence intervals. *HRQoL* health-related quality of life, *AP* academic performance. *β*_CL-1_ the cross-lagged path 1, where HRQoL score at baseline predicts AP at follow-up; *β*_CL-2_ the cross-lagged path 2, where AP at baseline predicts HRQoL score at follow-up; *β*_CS-Baseline_ the cross-sectional association between HRQoL and AP within baseline; *p*_FDR_ significant levels adjusted for multiple testing. *CFI* comparative fit index, *RMSEA* root mean square error of approximation, *GPA* grade point average. Cross-lagged models were adjusted for sex, pubertal stage, waist circumference, socioeconomic status, and parents’ education level. Statistically significant values are shown in bold

Autoregressive coefficients are presented in Table S1. The coefficient for HRQoL was small (β ranging from 0.458 to 0.487), while the autoregressive coefficient for academic performance indicators was excepting for physical education (*β* = 0.376) close to one (β ranging from 0.622 to 0.807).

**Fig. 2:**
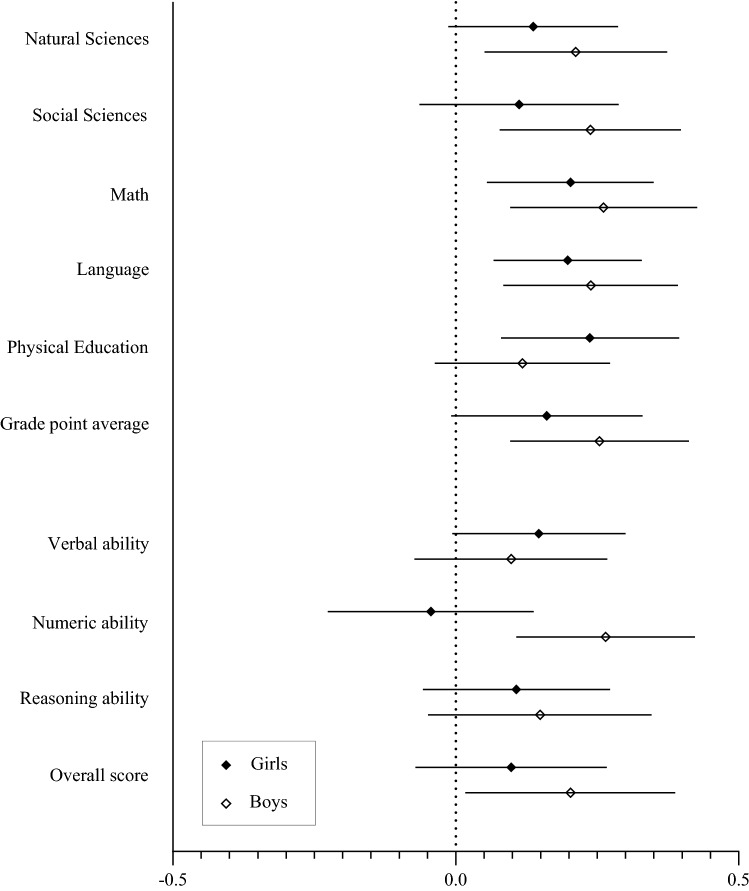
Stratified analyses by sex for the cross-lagged path in which academic performance at baseline predicts health-related quality of life at follow-up. Results showed as standardized coefficients and 95% confidence intervals. Analyses were adjusted for pubertal stage, waist circumference, socioeconomic status, and parents’ education level. Girls: *n* = 121, boys: *n* = 136

Sensitivity analyses were conducted with the sample reduced to those with complete HRQoL and academic performance data at both timepoints (*n* = 184), and results were largely similar (Tables S2 and S3). In addition, stratified analyses by sex for the cross-lagged path in which academic performance at baseline predicts HRQoL at follow-up (β_CL-2_) are presented in Fig. 2. In girls, math, language, physical education, and GPA at baseline were positively associated with HRQoL at follow-up, while in boys, all the academic grades indicators (except physical education), numeric ability, and the overall score of academic abilities at baseline were positively associated with HRQoL 24 months later.

## Discussion

The main findings of the present study indicated that HRQoL at baseline did not predict academic performance 24 months later, while all the academic grades and the overall score of academic abilities at baseline predicted HRQoL at follow-up in adolescents. Results of the stratified analyses by sex were largely similar. Specifically, in girls, math, language, physical education, and GPA at baseline predicted HRQoL 24 months later, while in boys, all the academic grades indicators (except physical education), numeric ability, and the overall score of academic abilities at baseline predicted HRQoL at follow-up. The current study contributes to the existing literature by exploring how HRQoL and a wide range of academic performance indicators are related to each other across time.

To our knowledge there is only one previous study analyzing the bidirectional association between HRQoL and academic performance in youth [15], which hampers further comparisons with other studies. In this study, Bortes et al. [15] analyzed a sample of 723 Swedish adolescents (15 years at baseline) showing a reciprocal relationship between HRQoL and GPA in girls, but not in boys. Importantly, the bidirectional associations found in girls did not point in the same directions, that is to say, HRQoL at baseline was positively associated with GPA 24 months later, while GPA at baseline was negatively associated with HRQoL at follow-up. Although these results differ from those found in the present study, our findings partially concur with prior evidence analyzing the bidirectional association between the broad higher-order construct of well-being and academic performance. Particularly, Steinmayr et al. [13] examined the bidirectional association between subjective well-being (including cognitive and affective components) and GPA in 290 German adolescents. In line with our results, the authors concluded that GPA at baseline was positively associated with changes in life satisfaction (the cognitive component of well-being) at 1-year follow-up. Similarly, one study conducted in 807 Chinese children (9 years) revealed that academic performance positively predicted subjective well-being 18 months later [27]. Likewise, Ng et al. [14] suggested that adolescents’ GPA exerted a positive effect on their life satisfaction 5 months later. However, contrarily to our findings, they also indicated that adolescents’ life satisfaction, albeit to a lesser extent, exerted a positive effect on their subsequent GPA. Also in contrast to our results, Wu et al. [12] reported that life satisfaction together with positive affect positively predicted adolescents’ academic performance (measured by a single index) 14 months later. Collectively, more longitudinal studies with longer designs and including the specific construct of HRQoL and multiple academic performance indicators are needed to elucidate the directionality of the HRQoL-academic performance association.

The reasons underlying why academic performance may positively predict HRQoL 24 months later cannot be elucidated in the present study; however, we suggest some mechanisms that may explain this association. First, based on the self-determination theory developed by Ryan and Deci in the seventies and eighties, academic performance may improve HRQoL through fulfilling the need for competence, which is considered a basic need that is essential for personal well-being [28]. Second, we speculate that health status could play an important role in this association. For instance, better academic performance may increase self-esteem with positive effects on well-being [27]. In addition, it is likely that adolescents with better academic performance are more health literate, which could lead them to take better care of themselves and have a better perception of their overall health status, positively influencing their HRQoL. Lastly, both academic performance and HRQoL may be influenced by diverse school-related variables. In fact, academic engagement, which is widely acknowledged as an important determinant of successful academic performance, has also shown to predict well-being [29], which may partially explain the academic performance-HRQoL association. In addition, although academic performance seems to be strongly linked to cognition, it also involves non-cognitive skills such as school environment, motivation, effort, attitude, interest, family support, personality, autonomy, self-perception, social acceptance, or teaching influence [30, 31], which in turn, may influence HRQoL.

Regarding stratified analyses by sex, some differences in both school performance and psychological-related issues between adolescent girls and boys could partially explain the sex-specific results found in the present study. In the case of physical education, which positively predicted HRQoL 24 months later in girls (but not in boys), we speculate that since girls tend to be more aware of their personal appearance and are more frequently dissatisfied by their body image than boys [32], girls could be specially interested in the health-related contents addressed in this subject, which in turn may positively influence their HRQoL. Meanwhile, the divergent results found for natural sciences, social sciences, and numeric ability, which were positively associated to HRQoL at follow-up in boys, but not in girls, could be explained not only by sex individual differences in terms of abilities, but also by the biological and cultural transmission of gender stereotypes [33]. In this sense, on the one hand, a previous study has stated that girls tend to achieve higher grades in subjects including emotional involvement (e.g., subjects related to humanities and social sciences) because of teachers' expectancies [33]. On the other hand, the belief that boys should get higher grades in mathematics than girls may increase their self-efficacy and self-concept towards this ability [33], which in turn may positively influence their HRQoL. Collectively, these issues and the fact that girls’ HRQoL declines more than boys’ over time [4, 34] may partially explain the sex-differences found in this longitudinal study.

Our findings have several important implications from an educational and public health perspective. In this sense, according to the OECD [35], ‘‘academic achievement that comes at the expense of students’ well-being is not a full accomplishment” (p. 4). Therefore, based on our results, policy makers should consider factors that influence adolescents’ academic performance to improve their HRQoL. In this context, education and health professionals should not only focus on study skills and pedagogy to improve adolescents’ academic performance, but also for instance on the promotion of healthy habits. Specifically, the promotion of compliance with healthy lifestyles (e.g., sleep, physical activity or diet) which have been positively associated with academic performance [36, 37] and the avoidance of unhealthy ones (e.g., screen time) which have been negatively associated with academic performance [38] could improve both their educational outcomes and their HRQoL.

### Limitations and strengths

Limitations of the study comprise the fact that data were collected only at two time points. Future research should include longitudinal data collected at various points in time to provide further evidence of the temporal precedence between HRQoL and academic performance. Yet, this study has several strengths, including the use of longitudinal data that enables to focus on changes over time rather than on static relations, as well as the analyses of a wide range of academic performance indicators, including individual academic grades and abilities. In addition, our statistical analyses were controlled for sex, pubertal stage, socioeconomic variables, and waist circumference which are relevant given their association with HRQoL and academic performance [6, 19, 20].

### Conclusions

In conclusion, our findings reveal that adolescents’ academic performance was positively associated with HRQoL 24 months later, showing largely similar results in girls and boys. Health and education professionals could benefit from collaborating to achieve both improved academic performance and HRQoL in youth. Further larger longitudinal and interventional studies in adolescents are warranted to clarify the pathways by which academic performance is linked to HRQoL, as well as to corroborate the directionality of this association.

## Supplementary Information

Below is the link to the electronic supplementary material.

Supplementary file1 (DOCX 19 KB)

## Data Availability

The data used for the study were collected from the LIFE Research Group. There are some restrictions on these data and are not available to the public. Those interested in accessing these data should contact the LIFE Research Group, University Jaume I, Castellon, Spain.

## References

[1] Fernández-López JA, Fernández-Fidalgo M, Cieza A (2010). Los conceptos de calidad de vida, salud y bienestar analizados desde la perspectiva de la clasificación internacional del funcionamiento (CIF). Revista Espanola de Salud Publica.

[2] Lyubomirsky S, King L, Diener E (2005). The benefits of frequent positive affect: Does happiness lead to success?. Psychological Bulletin.

[3] DeSalvo KB, Bloser N, Reynolds K, He J, Muntner P (2006). Mortality prediction with a single general self-rated health question a meta-analysis. Journal of General Internal Medicine.

[4] Bisegger C, Cloetta B, Von Rueden U, Abel T (2005). Health-related quality of life: Gender differences in childhood and adolescence. Sozial- und Präventivmedizin.

[5] Qi S, Qin Z, Wang N, Tse LA, Qiao H, Xu F (2020). Association of academic performance, general health with health-related quality of life in primary and high school students in China. Health and Quality of Life Outcomes.

[6] Donnelly JE, Hillman CH, Castelli DM, Etnier JL, Lee S, Tomporowski P, Lambourne K, Szabo-reed AN (2016). Physical activity, fitness, cognitive function, and academic achievement in children: a systematic review. Medicine and Science in Sports and Exercise.

[7] French MT, Homer JF, Popovici I, Robins PK (2015). What you do in high school matters: high school GPA, educational attainment, and labor market earnings as a young adult. Eastern Economic Journal.

[8] Lê-Scherban F, Diez Roux AV, Li Y, Morgenstern H (2014). Does academic achievement during childhood and adolescence benefit later health?. Annals of Epidemiology.

[9] Solera-Sanchez A, Adelantado-Renau M, Moliner-Urdiales D, Beltran-Valls MR (2021). Health-related quality of life in adolescents: Individual and combined impact of health-related behaviors (DADOS study). Quality of Life Research.

[10] Solera-Sanchez A, Adelantado-Renau M, Moliner-Urdiales D, Beltran-Valls MR (2021). Individual and combined impact of physical fitness on health-related quality of life during adolescence: DADOS Study. European Journal of Sport Science.

[11] Amholt TT, Dammeyer J, Carter R, Niclasen J (2020). Psychological well-being and academic achievement among school-aged children: a systematic review. Child Indicators Research.

[12] Wu X, Gai X, Wang W (2020). Subjective well-being and academic performance among middle schoolers: A two-wave longitudinal study. Journal of Adolescence.

[13] Steinmayr R, Crede J, McElvany N, Wirthwein L (2016). Subjective well-being, test anxiety, academic achievement: Testing for reciprocal effects. Frontiers in Psychology.

[14] Ng ZJ, Huebner SE, Hills KJ (2015). Life satisfaction and academic performance in early adolescents: evidence for reciprocal association. Journal of School Psychology.

[15] Bortes C, Ragnarsson S, Strandh M, Petersen S (2021). The bidirectional relationship between subjective well-being and academic achievement in adolescence. Journal of Youth and Adolescence.

[16] Ravens-Sieberer U, Erhart M, Rajmil L, Herdman M, Auquier P, Bruil J, Power M, Duer W, Abel T, Czemy L, Mazur J, Czimbalmos A, Tountas Y, Hagquist C, Kilroe J (2010). Reliability, construct and criterion validity of the KIDSCREEN-10 score: A short measure for children and adolescents’ well-being and health-related quality of life. Quality of Life Research.

[17] Ravens-Sieberer, U., Gosch, A., Erhart, M., von Rueden, U., Nickel, J., Kurth, B. M., Duer, W., Fuerth, K., Czemy, L., Auquier, P., Simeoni, M. C., Robitail, S., Tountas, Y., Dimitrakaki, C., Czimbalmos, A., Aszmann, A., Kilroe, J., Keenaghan, C., Bruil, J., … Waters, E. (2006). *The Kidscreen questionnaires quality of life questionnaires for children and adolescents; handbook*. Pabst Science Publ.

[18] Thurstone, L. L., & Thurstone, T. G. (2004). *TEA Test de Aptitudes Escolares (Scholar Aptitudes Test)*. *TEA Ediciones S. A.* (11th ed., Vol. 77). Madrid.

[19] Svedberg P, Nygren JM, Staland-Nyman C, Nyholm M (2016). The validity of socioeconomic status measures among adolescents based on self-reported information about parents occupations, FAS and perceived SES; implication for health related quality of life studies. BMC Medical Research Methodology.

[20] Pogodina A, Rychkova L, Kravtzova O, Klimkina J, Kosovtzeva A (2017). Cardiometabolic risk factors and health-related quality of life in adolescents with obesity. Childhood Obesity.

[21] Tanner JM, Whitehouse RH (1976). Clinical longitudinal standards for height, weight, height velocity, weight velocity, and stages of puberty. Archives of Disease in Childhood.

[22] Currie C, Molcho M, Boyce W, Holstein B, Torsheim T, Richter M (2008). Researching health inequalities in adolescents : The development of the Health Behaviour in School-Aged Children (HBSC) Family Affluence Scale. Social Science & Medicine.

[23] Rosseel Y (2012). Lavaan: An R package for structural equation modeling. Journal of Statistical Software.

[24] Selig JP, Little TD, Laursen BP, Little TD, Card NA (2012). Autoregressive and Cross-Lagged Panel Analysis for Longitudinal Data. Handbook of Developmental Research Methods.

[25] Hu L-T, Bentler PM (1999). Cutoff criteria for fit indexes in covariance structure analysis: Conventional criteria versus new alternatives. Structural Equation Modeling: A Multidisciplinary Journal.

[26] Benjamini Y, Hochberg Y (1995). Controlling the false discovery rate: a practical and powerful approach to multiple testing. Journal of the Royal Statistical Society.

[27] Yang Q, Tian L, Huebner ES, Zhu X (2019). Relations among academic achievement, self-esteem, and subjective well-being in school among elementary school students: a longitudinal mediation model. School Psychology Quarterly.

[28] Ryan RM, Deci EL (2000). Self-determination theory and the facilitation of intrinsic motivation, social development, and well-being. American Psychologist.

[29] Datu JAD, King RB (2018). Subjective well-being is reciprocally associated with academic engagement: A two-wave longitudinal study. Journal of School Psychology.

[30] Petrides KV, Chamorro-Premuzic T, Frederickson N, Furnham A (2005). Explaining individual differences in scholastic behaviour and achievement. British Journal of Educational Psychology.

[31] Degoy E, Berra S (2018). Differences in health-related quality of life by academic performance in children of the city of Cordoba-Argentina. Quality of Life Research.

[32] Jimenez-Flores P, Jimenez-Cruz A, Bacardi-Gascon M (2017). Body-image dissatisfaction in children and adolescents: A systematic review. Nutricion Hospitalaria.

[33] Trusz S (2020). Why do females choose to study humanities or social sciences, while males prefer technology or science? Some intrapersonal and interpersonal predictors. Social Psychology of Education.

[34] Michel G, Bisegger C, Fuhr DC, Abel T (2009). Age and gender differences in health-related quality of life of children and adolescents in Europe: A multilevel analysis. Quality of Life Research.

[35] OECD. (2015). Do teacher-student relations affect students’ well-being at school? *PISA in Focus*, *50*, 1–4. Retrieved from https://www.oecd-ilibrary.org/docserver/5js391zxjjf1-en.pdf?expires=1649432865&id=id&accname=guest&checksum=8CB81156661C52521111379E18044A77.

[36] Adelantado-Renau M, Jiménez-Pavón D, Beltran-Valls MR, Moliner-Urdiales D (2019). Independent and combined influence of healthy lifestyle factors on academic performance in adolescents: DADOS Study. Pediatric Research.

[37] Álvarez-Bueno C, Pesce C, Cavero-Redondo I, Sánchez-López M, Garrido-Miguel M, Martínez-Vizcaíno V (2017). Academic achievement and physical activity: a meta-analysis. Pediatrics.

[38] Adelantado-Renau M, Moliner-Urdiales D, Cavero-Redondo I, Beltran-Valls MR, Martínez-Vizcaíno V, Álvarez-Bueno C (2019). Association between screen media use and academic performance among children and adolescents. A systematic review and meta-analysis. JAMA Pediatrics.

