# Supplementary material for: The bidirectional longitudinal association between health-related quality of life and academic performance in adolescents: DADOS study
Source: Qual Life Res. 2022 Nov 16;32(3):729–38. doi: 10.1007/s11136-022-03291-z (PMC9992255; doi:10.1007/s11136-022-03291-z)
Supplement: Supplementary file 1 — Supplementary file1 (DOCX 19 KB) [file 11136_2022_3291_MOESM1_ESM.docx]

| **Table S1**  Autoregressive associations and complete overview of fit indices of cross-lagged panel models estimating the bidirectional associations between health-related quality of life and academic performance (N = 257). | | | | | | | | |
| --- | --- | --- | --- | --- | --- | --- | --- | --- |
|  | Autoregressive | | | | | | Fit indices | |
|  | β_AR-Health-related quality of life_ | | *p* | β_AR-Academic performance_ | | *p* | TLI | SRMR |
| **Health-related quality of life** | | | | | | | | |
| *Academic grades* | | | | | | | | |
| Natural Sciences |  | 0.471 (0.363, 0.580) | **<0.001** |  | 0.701 (0.632, 0.771) | **<0.001** | 0.908 | 0.031 |
| Social Sciences |  | 0.468 (0.359, 0.577) | **<0.001** |  | 0.626 (0.536, 0.716) | **<0.001** | 0.964 | 0.029 |
| Math |  | 0.458 (0.348, 0.569) | **<0.001** |  | 0.645 (0.557, 0.732) | **<0.001** | 0.930 | 0.030 |
| Language |  | 0.472 (0.367, 0.577) | **<0.001** |  | 0.667 (0.581, 0.753) | **<0.001** | 0.908 | 0.032 |
| Physical Education |  | 0.472 (0.365, 0.578) | **<0.001** |  | 0.376 (0.258, 0.495) | **<0.001** | 1.074 | 0.020 |
| GPA |  | 0.462 (0.354, 0.569) | **<0.001** |  | 0.807 (0.748, 0.866) | **<0.001** | 0.936 | 0.027 |
| *Academic abilities* | | | | | | | | |
| Verbal ability |  | 0.477 (0.368, 0.585) | **<0.001** |  | 0.656 (0.577, 0.736) | **<0.001** | 1.013 | 0.021 |
| Numeric ability |  | 0.468 (0.358, 0.578) | **<0.001** |  | 0.714 (0.638, 0.790) | **<0.001** | 0.910 | 0.026 |
| Reasoning ability |  | 0.487 (0.382, 0.593) | **<0.001** |  | 0.622 (0.521, 0.723) | **<0.001** | 0.997 | 0.026 |
| Overall score |  | 0.473 (0.366, 0.580) | **<0.001** |  | 0.774 (0.707, 0.840) | **<0.001** | 0.964 | 0.022 |
| Results showed as standardized coefficients and 95% confidence intervals. β_AR-Health-related quality of life_ = the autoregressive coefficient for health-related quality of life; β_AR-Academic performance_ = the autoregressive coefficient for academic performance. TLI = Tucker-Lewis index, SRMR = standardized root mean square residual, GPA = grade point average. Models were adjusted for sex, pubertal stage, waist circumference, socioeconomic status, and parents’ education level. Statistically significant values are shown in bold. | | | | | | | | |

| **Table S2**  Bidirectional associations between health-related quality of life and academic performance based on the cross-lagged panel models of the complete cases (N = 184). | | | | | | | | | | | | | | |
| --- | --- | --- | --- | --- | --- | --- | --- | --- | --- | --- | --- | --- | --- | --- |
|  | HRQoL → AP | | | | AP → HRQoL | | | | Cross-sectional | | | | Fit measures | |
|  | β_CL-1_ | | *p* | *p* _FDR_ | β_CL-2_ | | *p* | *p* _FDR_ | β_CS-Baseline_ | | *p* | *p* _FDR_ | CFI | RMSEA |
| **Health-related quality of life** | | | | | | | | | | | | | | |
| *Academic grades* | | | | | | | | | | | | | | |
| Natural Sciences |  | 0.003 (-0.095, 0.101) | 0.952 | 0.952 |  | 0.189 (0.085, 0.294) | **0.001** | **0.003** |  | 0.107 (-0.016, 0.231) | 0.094 | 0.213 | 0.959 | 0.071 |
| Social Sciences |  | 0.080 (-0.049, 0.208) | 0.222 | 0.555 |  | 0.192 (0.073, 0.311) | **0.002** | **0.004** |  | 0.108 (-0.018, 0.234) | 0.101 | 0.213 | 0.986 | 0.038 |
| Math | - | 0.128 (-0.239, -0.016) | **0.023** | 0.230 |  | 0.254 (0.144, 0.363) | **<0.001** | **<0.001** |  | 0.100 (-0.022, 0.222) | 0.109 | 0.213 | 0.968 | 0.059 |
| Language | - | 0.041 (-0.151, 0.068) | 0.464 | 0.601 |  | 0.239 (0.138, 0.341) | **<0.001** | **<0.001** |  | 0.096 (-0.020, 0.213) | 0.103 | 0.213 | 0.955 | 0.072 |
| Physical Education |  | 0.111 (-0.031, 0.254) | 0.140 | 0.540 |  | 0.153 (0.043, 0.264) | **0.005** | **0.008** |  | 0.103 (-0.029, 0.235) | 0.128 | 0.213 | 1.000 | 0.000 |
| GPA | - | 0.017 (-0.103, 0.070) | 0.709 | 0.788 |  | 0.222 (0.111, 0.334) | **<0.001** | **<0.001** |  | 0.138 (0.025, 0.251) | **0.018** | 0.180 | 0.970 | 0.069 |
| *Academic abilities* | | | | | | | | | | | | | | |
| Verbal ability | - | 0.056 (-0.177, 0.064) | 0.365 | 0.601 |  | 0.131 (0.012, 0.251) | **0.032** | **0.040** |  | 0.054 (-0.096, 0.203) | 0.478 | 0.531 | 0.999 | 0.011 |
| Numeric ability | - | 0.079 (-0.192, 0.0339 | 0.162 | 0.540 |  | 0.118 (-0.021, 0.258) | 0.091 | 0.091 |  | 0.075 (-0.057, 0.208) | 0.268 | 0.383 | 0.965 | 0.068 |
| Reasoning ability |  | 0.040 (-0.070, 0.150) | 0.481 | 0.601 |  | 0.117 (-0.010, 0.244) | 0.075 | 0.083 |  | 0.014 (-0.144, 0.171) | 0.866 | 0.866 | 1.000 | 0.000 |
| Overall score | - | 0.041 (-0.135, 0.052) | 0.385 | 0.601 |  | 0.154 (0.025, 0.284) | **0.019** | **0.027** |  | 0.057 (-0.096, 0.209) | 0.464 | 0.531 | 0.987 | 0.042 |
| Results showed as standardized coefficients and 95% confidence intervals. HRQoL = health-related quality of life, AP = academic performance. β_CL-1_ = the cross-lagged path 1, where HRQoL score at baseline predicts AP at follow-up; β_CL-2_ = the cross-lagged path 2, where AP at baseline predicts HRQoL score at follow-up; β_CS-Baseline_ = the cross-sectional association between HRQoL and AP within baseline; *p* _FDR_ = significant levels adjusted for multiple testing. CFI = comparative fit index, RMSEA = root mean square error of approximation, GPA = grade point average. Cross-lagged models were adjusted for sex, pubertal stage, waist circumference, socioeconomic status, and parents’ education level. Statistically significant values are shown in bold. | | | | | | | | | | | | | | |

| **Table S3**  Autoregressive associations and complete overview of fit indices of cross-lagged panel models estimating the bidirectional associations between health-related quality of life and academic performance of the complete cases (N = 184). | | | | | | | | |
| --- | --- | --- | --- | --- | --- | --- | --- | --- |
|  | Autoregressive | | | | | | Fit indices | |
|  | β_AR-Health-related quality of life_ | | *p* | β_AR-Academic performance_ | | *p* | TLI | SRMR |
| **Health-related quality of life** | | | | | | | | |
| *Academic grades* | | | | | | | | |
| Natural Sciences |  | 0.489 (0.378, 0.600) | **<0.001** |  | 0.689 (0.617, 0.762) | **<0.001** | 0.893 | 0.031 |
| Social Sciences |  | 0.487 (0.376, 0.598) | **<0.001** |  | 0.617 (0.524, 0.710) | **<0.001** | 0.963 | 0.030 |
| Math |  | 0.480 (0.367, 0.592) | **<0.001** |  | 0.623 (0.534, 0.712) | **<0.001** | 0.916 | 0.031 |
| Language |  | 0.493 (0.386, 0.600) | **<0.001** |  | 0.654 (0.565, 0.743) | **<0.001** | 0.883 | 0.033 |
| Physical Education |  | 0.491 (0.381, 0.600) | **<0.001** |  | 0.381 (0.266, 0.497) | **<0.001** | 1.079 | 0.020 |
| GPA |  | 0.481 (0.372, 0.591) | **<0.001** |  | 0.792 (0.728, 0.857) | **<0.001** | 0.922 | 0.028 |
| *Academic abilities* | | | | | | | | |
| Verbal ability |  | 0.495 (0.384, 0.606) | **<0.001** |  | 0.651 (0.567, 0.735) | **<0.001** | 0.997 | 0.024 |
| Numeric ability |  | 0.487 (0.374, 0.600) | **<0.001** |  | 0.712 (0.632, 0.793) | **<0.001** | 0.908 | 0.028 |
| Reasoning ability |  | 0.505 (0.396, 0.613) | **<0.001** |  | 0.612 (0.513, 0.711) | **<0.001** | 1.018 | 0.026 |
| Overall score |  | 0.491 (0.381, 0.601) | **<0.001** |  | 0.763 (0.694, 0.832) | **<0.001** | 0.966 | 0.024 |
| Results showed as standardized coefficients and 95% confidence intervals. β_AR-Health-related quality of life_ = the autoregressive coefficient for health-related quality of life; β_AR-Academic performance_ = the autoregressive coefficient for academic performance. TLI = Tucker-Lewis index, SRMR = standardized root mean square residual, GPA = grade point average. Models were adjusted for sex, pubertal stage, waist circumference, socioeconomic status, and parents’ education level. Statistically significant values are shown in bold. | | | | | | | | |
